# The Sanitarian

**Published:** 1895-03

**Authors:** 


### The Sanitarian.

This very valuable journal on sanitation, now in its twenty-third year, is doing grand work in the cause of sanitary science; and will continue in the future, as it has been hitherto, devoted to the promotion of the art and science of sanitation, mentally and physically in all their relations; by the investigation, presentation and discussion of all subjects in this large domain, as related to personal and household hygiene, domicile, soil and climate, food and drink, mental and physical culture, habit and exercise, occupation, vital statistics, sanitary organizations and laws, in short everything promotive of, or in conflict with health, with the purpose of rendering sanitation a popular theme of study and universally practical.

The *Sanitarian* will contain ninety-six pages monthly, making two volumes during the year. The volumes begin January and July. Subscriptions are received at any time. The subscription price is $4 per year. All correspondence and exchanges should be sent to Dr. A. N. Bell, Brooklyn, N. Y.

